# Contrasting genomic properties of free-living and particle-attached microbial assemblages within a coastal ecosystem

**DOI:** 10.3389/fmicb.2013.00120

**Published:** 2013-05-30

**Authors:** Maria W. Smith, Lisa Zeigler Allen, Andrew E. Allen, Lydie Herfort, Holly M. Simon

**Affiliations:** ^1^Center for Coastal Margin Observation and Prediction, Oregon Health and Science UniversityBeaverton, OR, USA; ^2^Microbial and Environmental Genomics, J. Craig Venter InstituteSan Diego, CA, USA; ^3^Division of Environmental and Biomolecular Systems, Department of Science and Engineering, Oregon Health and Science UniversityBeaverton, OR, USA

**Keywords:** metagenome analysis, Columbia River coastal margin, environmental water, microbial communities, particle-attached and free-living microbes

## Abstract

The Columbia River (CR) is a powerful economic and environmental driver in the US Pacific Northwest. Microbial communities in the water column were analyzed from four diverse habitats: (1) an estuarine turbidity maximum (ETM), (2) a chlorophyll maximum of the river plume, (3) an upwelling-associated hypoxic zone, and (4) the deep ocean bottom. Three size fractions, 0.1–0.8, 0.8–3, and 3–200 μm were collected for each habitat in August 2007, and used for DNA isolation and 454 sequencing, resulting in 12 metagenomes of >5 million reads (>1.6 Gbp). To characterize the dominant microorganisms and metabolisms contributing to coastal biogeochemistry, we used predicted peptide and rRNA data. The 3- and 0.8-μm metagenomes, representing particulate fractions, were taxonomically diverse across habitats. The 3-μm size fractions contained a high abundance of eukaryota with diatoms dominating the hypoxic water and plume, while cryptophytes were more abundant in the ETM. The 0.1-μm metagenomes represented mainly free-living bacteria and archaea. The most abundant archaeal hits were observed in the deep ocean and hypoxic water (19% of prokaryotic peptides in the 0.1-μm metagenomes), and were homologous to *Nitrosopumilus maritimus* (ammonia-oxidizing Thaumarchaeota). Bacteria dominated metagenomes of all samples. In the euphotic zone (estuary, plume and hypoxic ocean), the most abundant bacterial taxa (≥40% of prokaryotic peptides) represented aerobic photoheterotrophs. In contrast, the low-oxygen, deep water metagenome was enriched with sequences for strict and facultative anaerobes. Interestingly, many of the same anaerobic bacterial families were enriched in the 3-μm size fraction of the ETM (2–10X more abundant relative to the 0.1-μm metagenome), indicating possible formation of anoxic microniches within particles. Results from this study provide a metagenome perspective on ecosystem-scale metabolism in an upwelling-influenced river-dominated coastal margin.

## Introduction

Our study site encompasses an upwelling-influenced river-dominated coastal margin of the Pacific Northwest region of the United States. The Columbia River (CR) and its tributaries are the largest source of freshwater, by volume, to the California Current System. The CR estuary has been characterized as a “bioreactor” for microbially driven transformations of organic carbon and nitrogen from both the river and the coastal ocean. These activities are enhanced by development of large and well-defined estuarine turbidity maxima (ETM) (Small et al., 1990; Baross et al., 1994; Crump et al., 1998, 1999; Small and Prahl, 2004). ETM events, which are the result of tidally modulated, hydrodynamic trapping of suspended particulate matter at the fresh-salt water interface, extend the particle residence time and provide “fertile grounds” for development of productive particle-associated estuarine microbial communities (Crump et al., 1998, 1999, 2004).

Bacterial carbon production measurements indicated much higher activities for particle-attached, compared to free-living, microbiota (both freshwater and estuarine samples) (Crump et al., 1998). Furthermore, particle-attached bacteria comprise up to 50% of total bacteria in the CR ETM. The highest measured production rates occurred at intermediate salinities, reflecting the critical roles estuarine microorganisms play in detrital processing before its export to the coastal ocean. Additionally, extracellular enzyme activity was found to be largely associated with particles and increased with turbidity. Smaller (3–10 μm in size), slow-settling particles were the most bacterially active, and are thought to be retained in the estuary through processes in the water column leading to larger, rapidly-settling macro-aggregates (Crump and Baross, 2000b).

Linking the CR to the Northeastern Pacific coastal ocean is the massive CR plume, in which estuarine water is tidally entrained and which, with seasonal variation, reaches dozens to hundreds of kilometers from the river mouth, providing a major source of nutrients to the continental shelf (Aguilar-Islas and Bruland, 2006; Bruland et al., 2008). The river and ocean end members create strong seasonal and inter-annual variability in the region, and the continuum between the river, estuary, plume, and host continental shelf is referred to hereafter as the Columbia River coastal margin (CRCM).

Another major source of nutrients to the CRCM is the nearshore seasonal upwelling that is frequently observed in summer when northerly winds push the surface water away from the coastline (Kudela et al., 2005). This offshore moving water is replaced by cooler, saltier and nutrient-rich water upwelling from depths of 150 to 300 m of the northward-flowing California Current Undercurrent (Roegner et al., 2011) to the upper euphotic water layers, resulting in increased primary production and phytoplankton blooms (Kudela et al., 2005). As a consequence, the decomposition of settling dead bloom biomass by aerobic heterotrophs leads to the formation of hypoxic zones with oxygen levels ≤2 mg L-1 (Rabalais et al., 2002; Roegner et al., 2011).

In earlier studies evaluating CR particle-attached microbiota, sequencing of small-subunit (SSU) ribosomal RNA (rRNA) genes indicated that most organisms were related to members of the genus Cytophaga or of the α, γ, or δ subclass of the Proteobacteria (Crump et al., 1999; Crump and Baross, 2000b). More recently, 454 pyrosequencing of PCR-amplified 16S rRNA genes was performed to examine bacteria community composition in >300 samples collected across diverse CRCM habitats over four years (2007–2010) (Fortunato et al., 2011). In addition to illuminating patterns of spatial and temporal variability in highly diverse CRCM microbial assemblages, results supported previous research (Crump et al., 1999; Crump and Baross, 2000a) in finding that free-living estuarine communities were not uniquely estuarine, but instead were comprised of taxa from the river and the coastal ocean. This result contrasts to previous research showing that particle-attached communities in the estuary were unique from those of either end-member population. In this study, we use metagenome-scale analysis to further test the hypothesis that properties related to organic matter processing in the CR are distinctive for particle-attached and free-living microbial assemblages. To address this question, we examined taxonomic and metabolic properties of size-fractionated metagenomes from water samples representing contrasting habitats (surface/deep, estuarine/marine) and lifestyles (heterotrophic/autotrophic) in the CR estuary and adjacent coastal Pacific Ocean.

## Materials and methods

### Water sample collection

**Figure 1:**
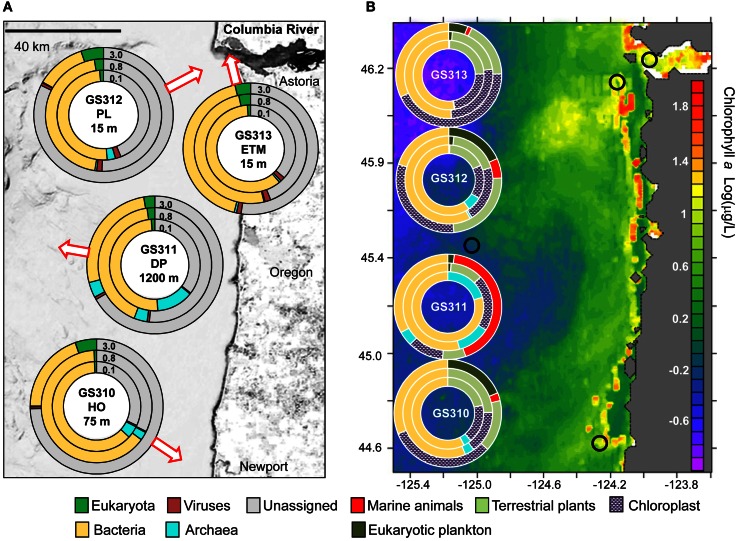
**Domain composition of metagenomes—arrows indicate approximate water sampling locations upon the underlying contour map.** Inner 0.1, middle 0.8, and outer 3.0 circles represent metagenomes from 0.1–0.8, 0.8–3.0, and 3–200 μm size fractions, respectively. **(A)** Domain abundance was calculated from predicted peptide annotations (hits with ≥30% identity for ≥70% of the alignment length) as percentages of all post-QC reads. **(B)** Domain abundance was calculated from SSU rRNA composition (% of total number of SSU rRNA reads). Small black circles indicate sampling locations mapped upon surface chlorophyll a Log(μg/L) in the northeastern Pacific coastal ocean southward from the Columbia River mouth (NOAA satellite data averaged over a period of 14 days centered around Aug 23, 2007).

Four water samples of 200–250 L each were collected during two CMOP research cruises on board the R/V Wecoma and the R/V Barnes in August 2007 using either 20 L Niskin bottles mounted to a CTD (conductivity-temperature-depth meter) rosette (GS310, 311, and 312), or an air pump with tubing for the estuarine sample GS313. Sampling locations are shown in the map in Figure 1. To collect three different size fractions, the environmental water was pre-filtered with a 200-μm nytex net, followed by serial filtration through 3-, 0.8-, and 0.1-μm Supor 293 mm disc filters (Pall Life Sciences, Ann Arbor, MI, USA).

### Environmental data

Table 1 shows environmental data associated with the water samples. Salinity (PSU), temperature (°C), oxygen (mg L-1), and turbidity (NTU) were recorded during sample collection with a Sea-Bird 911+ CTD (conductivity-temperature-depth) profiler (Sea-Bird Electronics Inc., Bellevue, WA). Aliquots of each water sample were collected to measure concentrations of macronutrients (nitrite + nitrate – referred to hereafter as nitrate, ammonium, silicic acid and phosphate) on a Lachat QuikChem 8000 Flow Injection Analysis system using standard colorimetric methods (Parsons et al., 1984). For photosynthetic pigment (chlorophyll a and phaeophytin a) analysis, suspended particulate matter was collected by filtering 100–300 mL of water onto a GFF filter (ø 25 mm, Whatman, Piscataway, NJ) and analyzed by high performance liquid chromatography (HPLC) as previously described (Herfort et al., 2011a). Percentages of photosynthetically active chl a were calculated as 100 × [chl a] / ([chl a] + [phaeophytin a]) (Herfort et al., 2011b). Dissolved organic carbon (DOC) particulate organic carbon (POC) and nitrogen (PON) were measured as described (Smith et al., 2010). Near real-time satellite data on ocean surface temperature and surface chl a (Figure 1) concentration for a 14-d period centered around August 23, 2007, were obtained from Live Access Server of Ocean Watch (The CoastWatch West Coast Regional Node, http://las.pfeg.noaa.gov/oceanWatch/oceanwatch.php). Calculations of the Coastal Upwelling Index (at 45° North) were performed in the CMOP Climatological Atlas as described (http://www.stccmop.org/datamart/virtualcolumbiariver/simulationdatabases/climatologicalatlas).

**Table 1:** **Environmental metadata and sequencing statistics**.

**Sample ID**	**GS310-HO**	**GS311-DP**	**GS312-PL**	**GS313-ETM**
Date	16 Aug 2007	17 Aug 2007	18 Aug 2007	28 Aug 2007
Habitat type	Hypoxic	Deep ocean	Plume chl max	ETM
Location^*^	NH10	CMa40	CR7	Mouth
Latitude (°)	44.651	45.483	46.167	46.250
Longitude (°)	−124.295	−124.918	−124.158	−123.986
Depth (m)	75	1200	15	15
Salinity (PSU)	33.93	34.45	31.47	19.05
Temperature (°C)	7.4	3.3	15.4	15.4
Oxygen (mg/L)	0.7	0.61	9.05	6.56
Turbidity (NTU)	2.95	2.08	2.71	19.25
PO_4_ (μM)	3.09	3.3	0.33	0.87
TDP (μM)	3.43	3.33	0.68	1.2
NO_3_ +NO_2_ (μM)	34.94	43.76	2.78	12.11
NH_4_ (μM)	0.44	0.16	0.48	1.22
TDN (μM)	42.8	49	12.5	22.1
Chlorophyll a (μg/L)	5.15	0	14.72	3.14 ± 0.61^*^
Phaeophytin a (μg/L)	1.83	0	0.32	0.26 ± 0.09^*^
Photosynthetically active chl a (%)^**^	74	0	98	90
DOC (mg/L)	1.6	1.44	1.16	1.85
POC (μg/L)	237.5	44.7	415.2	1667.5
PON (μg/L)	43.1	3	66	215
POC/PON Ratio	5.5	14.90	6.29	7.76
**0.1–0.8 μm FRACTION**				
Number of reads	663,013	710,477	587,204	643,999
Post-QC reads	530,848	561,066	478,603	484,724
% Annotated	81.42	80.19	63.15	78.52
GC content (%)	38	39	37	42
Genome size (Mbp)	1.30	1.04	0.99	1.30
**0.8–3 μm FRACTION**				
Number of reads	275,758	316,062	243,707	149,711
Post-QC reads	246,287	287,577	219,720	130,255
% Annotated	80.62	56.01	55.34	69.29
GC content (%)	42	49	49	50
Genome size (Mbp)	1.48	1.33	1.59	1.85
**3–200 μm FRACTION**				
Number of reads	296,862	598,827	287,012	215,849
Post-QC reads	225,654	512,566	198,156	185,969
% Annotated	31.70	42.10	24.71	59.54
GC content (%)	47	50	49	48
Genome size (Mbp)	1.38	1.66	2.09	1.83

Sample names are composed of the GS (“global survey”) number of the JCVI sample database and habitat: HO, hypoxic water; DP, deep ocean bottom; PL, plume; ETM, estuarine turbidity maxima. Sampling locations are encoded as follows: NH10, Newport Hydroline, 10 km from the coast; CMa40, Cape Meares, 40 km offshore; CR7, Columbia River transect, 7 km offshore; Mouth, Columbia River mouth. Each sample was pre-filtered with a 200 μm net, and fractionated using three sequential collection filters, 3, 0.8, and 0.1 μm.

* *For the GS313 sample, water was not collected for pigment analysis. However, pigment analysis was done for seven samples collected from bottom ETM events within 24 h of the GS313 sample collection. Average values (plus/minus standard deviations) for those samples are provided for GS313*.

** *Calculated as (100 × [chl a])/([chl a] + [pheo])*.

### DNA extraction and 454 pyrosequencing

The size-fractionated samples were stored at −80°C, and used for isolation of total environmental DNA (Allen et al., 2012). Total environmental DNA was sheared and adaptor-ligated, followed by emulsion PCR amplification, AMPure bead purification, and sequencing using the Roche 454 GS FLX Titanium platform at JCVI (Allen et al., 2012). Generally, a quarter-plate or a half-plate run was performed for individual size fractions of each sample.

### Data submission and accession numbers

The twelve 454 metagenomic datasets were submitted to the IMG/M-ER metagenome database with the submission IDs 6836–6846, and 8114 (sample IDs 1063–1073, and 1644) of the ER submission project 2387, “Marine, estuarine microbial communities from CRCM.” In addition, the data are deposited in CAMERA (Community Cyberinfrastructure for Advanced Microbial Ecology Research and Analysis, camera.calit2.net), accession number CAM_P_0001024.

### Sequence data processing and metagenome annotations

The 454-pyrosequencing reads were processed for quality control and removal of artificial replicates (sequences sharing >90% nucleotide identity beginning with the same three nucleotides) as described previously (Allen et al., 2012). Since high complexity of marine microbial communities precluded contig assembly, the processed reads were annotated as unassembled singletons by uploading to the Integrated Microbial Genomes with Microbiome Samples—Expert Review web server (IMG/M-ER of the DOE Joint Genome Institute, https://img.jgi.doe.gov/cgi-bin/mer/main.cgi). Table 1 shows the sequencing statistics data generated by the automated pipeline of IMG/M-ER. The IMG/M-ER server performed prediction of the most probable coding sequences (CDS), and non-coding RNA genes, and subsequent functional and taxonomic CDS annotations (Markowitz et al., 2008, 2012). Functional profiling was done (1) via homology (basic local alignment search tool, BLAST) to peptide sequence data of Clusters of Orthologous Groups of proteins (COGs), KEGG and MetaCyc pathways, Enzymes, Gene Ontology (GO) categories, or (2) via Hidden Markov models (HMM) approach to Pfam (protein families) 26.0 database.

### Analysis of peptide composition and protein recruitment plots

Peptide prediction was performed by the IMG/M-ER annotation pipeline using the *E*-value cutoff of 10^−5^ as the default. Based on previous studies (Rost, 1999; Konstantinidis and Tiedje, 2005), peptide annotations were also filtered using a cutoff of ≥30% amino acid sequence identity over ≥70% of the length of a pair-wise alignment for a given sequence read with the corresponding top hit reference. The two additional optional cutoffs, ≥60 and ≥90% identity (over ≥70% of the amino acid alignment length), provided for high stringency and high taxonomic resolution at family to genus, and genus to species levels, respectively. The ≥90% cutoff, while allowing distinction among species (Goris et al., 2007), decreased the numbers of annotated peptides drastically and was of value only for highly abundant and well-characterized microorganisms. These three cut-offs were also used by the IMG/M-ER protein recruitment plot tool that aligned homologous metagenome hits to a given reference genome (Rusch et al., 2007).

### Metagenome comparison and effective genome size estimation

Metagenomes were analyzed by similarity at taxonomic and functional levels. For a taxonomic category, i.e., a microbial family, the relative abundance value was calculated in a metagenome as the sum of all reads annotated to this taxon divided by the overall sum of post-QC sequence reads for the corresponding metagenome. In some cases (indicated in the text) the abundance values for bacterial families were expressed as percentages of total predicted bacterial peptide reads. Since the abundance values were expressed in percent, the significance of a difference between two given samples was calculated based on the two sample *t*-test between percentages. Relative abundance values of microbial taxa were used for comparison of the 12 metagenomes using hierarchical clustering and principal component analyses with the IMG/M-ER online tools, or with software programs Cluster and TreeView (Eisen et al., 1998). Functional comparison of metagenomes was done using IMG/M-ER normalized difference, or D-score and D-rank calculated as described (Markowitz et al., 2008), https://img.jgi.doe.gov/cgibin/mer/main.cgi?section=AbundanceComparisonsSub&page=dscoreNote.

Effective bacterial genome size was calculated by estimating the density of genome equivalents from a previously defined set of 35 core, mostly single-copy predicted bacterial marker proteins (Raes et al., 2007). The bacterial marker peptide density in a metagenome was normalized by the total number of bacterial peptides. The resultant effective genome sizes (Table 1) were used to calculate the number of bacterial genome equivalents for each metagenome. These values were then used to calculate the relative abundance values for functional gene categories of interest (COGs, Pfams, or Enzymes): the total number of hits for a category in a given size fraction was divided by the number of bacterial genome equivalents in the corresponding metagenome (Allen et al., 2012).

### Analyses of small subunit rRNA composition

Predicted SSU rRNA sequences were annotated using the CAMERA Portal with the rRNA Taxonomy Binning workflow. Two reference databases were used to analyze taxonomic composition of predicted SSU rRNA genes: Silva SSU r108 (http://www.arb-silva.de/) for both prokaryotic and eukaryotic SSU rRNA, and Greengenes (http://greengenes.lbl.gov/cgi-bin/nph-index.cgi) for prokaryotic rRNA composition. CAMERA-generated annotations were filtered using the 10^−30^ *E*-value cutoff. Predicted SSU rRNA counts ranged from 149 to 869 in individual metagenomes. For each metagenome, the taxonomic abundance of a phylogenetic type (phylotype) was calculated as a percentage of the total number of identified SSU rRNA reads.

To estimate the diversity within taxonomic groups, the SSU rRNA fragments corresponding to two groups of highly abundant microorganisms, Thaumarchaeota related to *Nitrosopumilus maritimus* SCM1, and Alphaproteobacteria of SAR11 clade, were de-replicated to <97% using Jalview (http://www.jalview.org/) (Waterhouse et al., 2009), and then aligned to the full-length SSU rRNA gene from a fully sequenced genome representative of these taxa using the CLC Free Workbench 4.6 (CLC Bio, Cambridge, MA). Number of OTUs for each microbial group was calculated as the maximum number of non-redundant fragments aligning to the full-length gene at any given location.

## Results

### Habitat and biogeochemical characteristics of sequenced water samples

Four large volume (>200 L) samples were collected for high-resolution metagenome sequencing from diverse locations in the CR estuary and adjacent ocean along the Oregon coast. Sample collections took place during two ship-based field campaigns in August 2007 (Figure 1A, Table 1). Late summer conditions in the region typically consist of: (1) low CR discharge, and low nutrient concentrations in river water, (2) highly stratified estuarine water column with prominent ETM, (3) low-volume, upwelling-dominated plume with high surface salinities, (4) upwelling-associated formation of hypoxic zones in close proximity to the coast, and (5) abundant phytoplankton blooms (Small et al., 1990; Prahl et al., 1997; Colbert and McManus, 2003; Hickey and Banas, 2003; Kudela et al., 2005; Bruland et al., 2008; Roegner et al., 2011). The environmental data collected in parallel with the samples during August 15–28, 2007 cruises were consistent with this description (Table 1). CMOP simulations indicated that strong upwelling during the two weeks prior to sample collection resulted in formation of a nearshore band of colder, high-salinity water prominent at 10 and 20 m depth adjacent to the coast and CR mouth. The simulations were consistent with the NOAA satellite observation data for the 2-week period around August 23rd, which demonstrated lower surface water temperatures along the coast. This band of nearshore upwelled water in the satellite data was associated with increased concentrations of surface chl a, in comparison with the open ocean, indicating phytoplankton bloom(s) (Figure 1B). Growth rates of heterotrophic plankton, and chl a and total RNA concentrations in >30 samples collected during the August 2007 cruise also indicated prominent plankton blooms occurring in the CRCM and the nearshore ocean (Smith et al., 2010; Herfort et al., 2011b).

The GS313-ETM sample was collected during an ETM event in the CR estuary close to the river mouth at 15 m depth. At that time, the average water residence in the estuary was estimated to be 8.5 d (data not shown), exceeding the bacterial doubling time of 10 h–2 days. Similar conditions were reported to promote the propagation of unique estuarine bacterial populations to high levels (Crump et al., 1998, 1999, 2004). Measurements of the GS313-ETM sample showed (1) high turbidity and POC and PON content; (2) relatively low dissolved nitrate and phosphate concentrations, and (3) relatively high oxygen content (due to intense mixing of the water column) (Table 1). The ammonium concentration was indicative of organic matter remineralization, which reaches high levels in the estuary in summer (Gilbert et al., 2013). The observed chl a content likely represented traces from a bloom of the mixotrophic ciliate, *Mesodinium rubrum*, that was observed nearby at the time of sampling (Herfort et al., 2011a, 2012). Detailed characterization of several additional ETM samples indicated they were “hotspots” of heterotrophic bacterial activity for transformation and degradation of organic matter from expired marine, freshwater, and estuarine phytoplankton (Herfort et al., 2011a, 2012), consistent with previous reports (Crump et al., 1998, 1999, 2004).

The GS312-PL sample was collected within a chlorophyll maximum of an aged, high salinity plume (Table 1). An estimation of 98% photosynthetically active chl a (indicating high chl a and low pheophytin concentrations), and high levels of oxygen, POC and PON collectively indicated that abundant, living phytoplankton were present at the time of sampling. Furthermore, despite observed nearshore upwelling, the nitrate concentration in the sample was very low (Table 1), likely because of consumption by the bloom.

Another bloom-associated sample, GS310-HO, was collected at 75 m depth from a hypoxic zone that formed on the shelf. Measurements of the sample showed (1) relatively high nitrate and phosphate concentrations, typical for nearshore upwelling, (2) significant levels of both chl a and pheophytin with only 74% of photosynthetically active chl a, suggestive of pigment degradation; and (3) relatively high levels of POC and PON (Table 1). Depleted oxygen and elevated ammonium concentrations suggested remineralization of a senescent phytoplankton bloom associated with GS310-HO.

Finally, the GS311-DP sample was collected in the adjacent deep ocean off of the shelf; close to the bottom at 1200 m. It was characterized by (1) low temperature, (2) low oxygen, (3) high nitrate and phosphate concentrations; (4) the absence of chl a, and (5) relatively low POC and PON (Table 1). This sample also had the highest POC:PON ratio, which may be indicative of DNA degradation within particulate organic matter (Herfort et al., 2011b).

### Size fraction metagenome features

For each sample, serial filtration was used to collect microorganisms: 0.1–0.8, 0.8–3, and 3-200-μm (designated as “0.1,” “0.8,” and “3.0”-μm size fractions, respectively). In total, 12 size fractions from four samples provided ~5 million reads – 4 million (1.8 Gbp) passed quality control and filtering (Table 1). Approximately 70% of the post-QC reads (1.27 Gbp) was predicted as CDS, including some information on taxonomy and/or function. For each metagenome, the effective genome size was estimated using only predicted bacterial peptides, resulting in values that ranged from 0.99 to 2.09 Mbp (Table 1). These were consistent with previous estimates for marine and coastal samples (Raes et al., 2007; Allen et al., 2012). In general, the effective genome size was smaller in the 0.1-μm size fractions compared to the larger size fractions, with the largest genome size (Table 1) observed in the 3-μm plume metagenome. These calculated values likely underestimated actual genome sizes (Raes et al., 2007; Konstantinidis et al., 2009), since only annotated bacterial peptides were considered, and unclassified CDS were not taken into account.

Average GC content decreased with decreasing effective genome size. The lowest values (36–37% G + C) were observed for 0.1-μm size fractions of hypoxic water and plume samples (Table 1). In general, GC content tended to be higher for the ETM size fractions, relative to corresponding size fractions of the oceanic samples (Table 1). Altogether, these results are consistent with previous observations in finding that reduced GC content is typical for organisms with smaller genome sizes living in oligotrophic marine environments (Giovannoni et al., 2005b), while higher GC content is observed within environments experiencing upwelling (Allen et al., 2012).

### Taxonomic composition of analyzed metagenomes

#### Eukaryota

**Figure 2:**
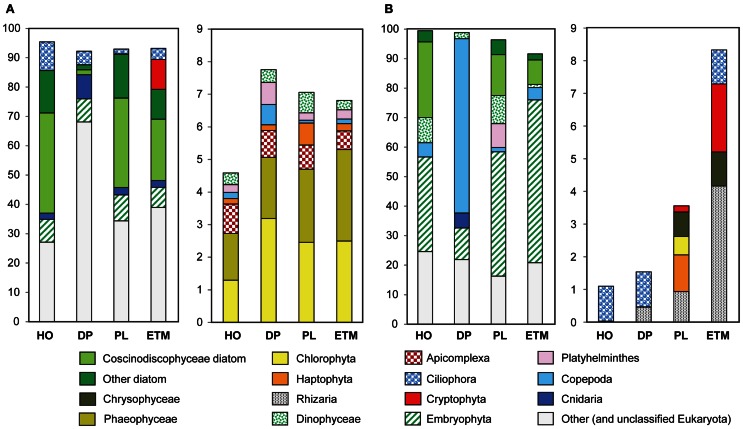
**Taxonomic composition of the domain Eukaryota in 3-μm metagenomes, indicated by habitats.** HO, hypoxic water; DP, deep ocean bottom; PL, plume; ETM, estuarine turbidity maxima. The left panel in both **(A)** and **(B)** represents taxon abundance for >90% of total, and the right panel shows the remaining 10% (only taxa with abundances above 0.5% threshold in at least one sample). Bars show taxon abundance in percentage of total, with color legend given below. **(A)** Eukaryotic peptide composition with abundance values for each taxon expressed as percentages of the corresponding hits (with ≥30% sequence identity over >70% of the alignment length) to the total predicted eukaryotic peptides. **(B)** Eukaryotic SSU rRNA composition with abundance values expressed as percentages of the corresponding 18S rRNA hits to the total eukaryotic SSU rRNA reads.

Despite the use of 200-μm nets for water prefiltering, significant numbers of reads were attributed to relatively large eukaryotic multicellular organisms, presumably from dead and decaying biomass. The highest abundance of Eukaryota was detected in the 3-μm fractions by predicted peptide reads (5–24% of all annotated peptides), and SSU rRNA reads (up to 44% of all SSU rRNA). The large numbers of rRNA cistronic repeats that are prevalent in eukaryotic genomes, and annotation artifacts caused by relatively high availability of 18S rRNA sequence information compared to fully sequenced genomes may have biased read abundances toward rRNA. Predicted peptide data is dependent upon the availability of reference genome information and may also be highly biased (Urich et al., 2008). Furthermore, 3-μm size fractions also contained the highest amounts of unclassified peptide sequences (Figure 1A, 63–82%), many of which may also be eukaryotic in origin. Thus, comparison of the two approaches produced some obvious discrepancies (Figure 2), especially for marine animals. Analysis of SSU rRNA showed enrichment of the deep ocean bottom metagenome with copepods (99% of which appeared similar to Calanoida); and of the plume metagenome with Platyhelminthes (flat worms) (Figure 2). The corresponding peptide data showed a very limited enrichment of copepods, and a different pattern of enrichment for flat worms (Figure 2).

Detailed analysis of Eukaryota is shown only for the 3-μm size fractions (Figure 2), because Eukaryotic annotations in 0.1- and 0.8-μm size fractions of all samples were dominated by vascular plant DNA (Embryophyta) that was likely terrestrial in origin (Figure 1B). These vascular plant sequences were observed in significant quantities: 7–12% of all annotated eukaryotic peptide reads, and 12–56% of all SSU rRNA (Figure 2). On average, the SSU rRNA fragments shared >98% sequence identity with the reference genomes. The most striking feature was a remarkably similar Embryophyta taxonomic composition across all habitats (data not shown). This result suggested relatively fast dispersal of lightweight, degradation-resistant pollen and spores over long distances, driven by wind and riverflow (Heusser and Balsam, 1977). Indeed, most sequences belonged to anemophilous families (such as Pinaceae, Poaceae, and Brassicaceae) or Bryopsida (mosses), whose pollen and spores, respectively, are produced, dispersed by wind, and deposited during late summer dry periods (Heusser and Balsam, 1977), coincident with our sample collections. This suggests that eukaryotic DNA from the soil and decaying terrestrial organic matter was quickly degraded in water, or possibly even on land before reaching the river. Previous analysis of lignin phenol content in the CR estuary also showed that organic matter in the ETM was dominated by riverine and marine phytoplankton with vascular plant debris comprising only a minor fraction (Prahl et al., 1997).

Eukaryotic plankton composition also varied across habitats and size fractions (Figure 2). In all three euphotic zone samples marine diatoms dominated phytoplankton taxa, mostly Coscinodiscophyceae that include Eucampia, Guinardia, Skeletonema, and Thalassiosira, with the highest relative abundance and species diversity observed in the coastal ocean hypoxic water and plume metagenomes (Figure 2). Samples from these two habitats were elevated in chl a content (Table 1), with correspondingly high surface chl a concentrations also observed in coastal satellite data around the time of sampling (Figure 1B). The associated water parameter data and low photosynthetically active chl a value (Table 1) (Herfort et al., 2011b) indicated that diatoms in the hypoxic zone sample were mostly dead, in contrast to the living diatoms in the plume. The ETM sample also contained marine diatoms, consistent with previous observations that these organisms provide a major source of labile particulate organic matter for microbial heterotrophs in the CR estuary (Herfort et al., 2011b). In contrast to metagenomes from the euphotic zone, only a very small amount of diatom-annotated peptides and no rRNA was observed in the deep ocean sample (Figure 2), suggesting degradation of diatom DNA before reaching ocean bottom.

Other eukaryotic taxa abundant in the euphotic zone samples were Dinophyceae (dinoflaggelates) and Ciliophora (ciliates), which may have been grazing on the diatom blooms and/or bloom-associated bacteria (Figure 2). Also abundant were (1) Haptophyta, with the highest abundance in the plume sample, and (2) Cryptophyta, mostly observed in the ETM, where up to 20% of all 16S rRNA genes represented Cryptophyta chloroplasts (Figure 2).

#### Archaea

**Figure 3:**
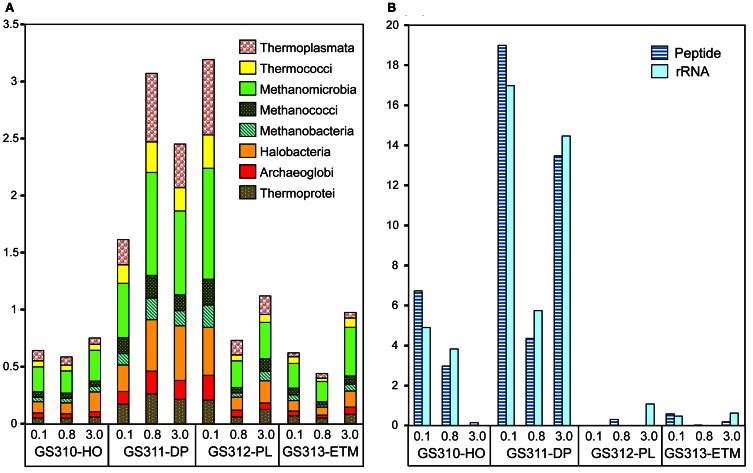
**Taxonomic composition of the domain Archaea sequences in metagenomes. (A)** Euryarchaeota and Crenarchaeota class abundance was calculated as a percentage of the corresponding hits (with ≥30% sequence identity over >70% of the alignment length) to the total predicted prokaryotic peptides. **(B)** Thaumarchaeota, Marine Group I, and *Nitrosopumilus maritimus*-like organism abundance was calculated either as percentage of total prokaryotic peptides at >60% identity (dark blue), or rRNA hits at >70% (light blue). Sample names are composed of the GS (“global survey”) number of the JCVI sample database and habitat: HO, hypoxic water; DP, deep ocean bottom; PL, plume; ETM, estuarine turbidity maximum. The numbers above the sample names indicate size fractions: 0.1, 0.1–0.8 μm; 0.8, 0.8–3 μm; 3, 0.8–200 μm.

The highest proportion of Archaea (14% of all predicted peptide reads) was observed in the 0.1-μm size fraction of the deep water sample, with significant numbers (3–5%) of reads observed in the particulate fractions as well (Figures 1A, 3A). Some of the taxa identified, i.e., Halobacteria (Euryarchaeota) include both aerobic and anaerobic members isolated from marine and estuarine water columns and sediments (e.g., Abreu et al., 2001). A relatively high proportion of total archaeal sequences (5–40% across metagenomes) were annotated to organisms considered to be strict anaerobes, such as methanogens, (i.e., Methanococci, Methanobacteria, Methanomicrobia, Figure 3A).

**Figure 4:**
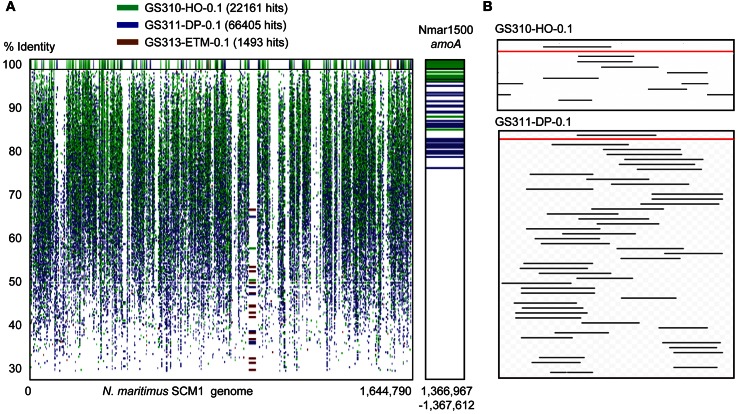
**(A)** Protein recruitment plot results showing metagenome peptide hits (with >30% identity over >70% of the alignment length) aligned against the homologous loci of the *Nitrosopumilus maritimus* SCM1 reference genome. The X-axis shows the hit position along the *N. maritimus* chromosome, and the Y-axis indicates the percent identity of a given sequence alignment for a metagenome hit and the reference locus, ranging from 30 to 100%. The upper panel displays the hits with 100% sequence identity. Metagenome hits from the hypoxic, deep, and ETM 0.1-μm size metagenomes are shown in green, blue and brown, respectively, with the corresponding hit numbers in brackets. The right panel is a blown up section of the recruitment plot containing the *N. maritimus* amoA gene encoding the active subunit of the archaeal ammonia monooxygenase enzyme. **(B)** Alignment of 16S rRNA hits (with dereplication to <97%) from two 0.1-μm metagenomes of the hypoxic (upper) and deep (lower) samples aligned against the *N. maritimus* 16S rRNA gene (the red lines).

Thaumarchaeota sequences were highly enriched in the 0.1-μm size fractions of the deep and hypoxic water samples (>66,000 and >22,000 peptide sequence hits, respectively; up to 19% of all predicted prokaryotic peptides) (Figure 3B). The corresponding peptides were mostly annotated to the family Nitrosopumilaceae, to Marine Group I (MGI) archaea related to “Candidatus *N. maritimus*” SCM1, which is one of the most abundant microbial groups contributing to oceanic nitrification in marine and coastal habitats (Schleper and Nicol, 2010). Protein recruitment plot analysis performed for metagenome data with the fully sequenced genome of SCM1 showed that the reference chromosome was tiled continuously with hits from two habitats: deep ocean bottom and hypoxic water (Figure 4A). The 0.1-μm metagenomes from both habitats showed approximately 1X coverage at >90% peptide sequence identity level, and 4X, and 14X coverage at 30–90% identity for the deep water and hypoxic fractions, respectively. These data suggest that presence of close relatives of the reference organism *N. maritimus* SCM1, as observed in other coastal samples (Tully et al., 2012). Protein recruitment indicated 63 and 55% average sequence similarity to the reference, respectively, for the hypoxic and deep water samples (*p* < 0.001). Consistent with this result, the deep ocean 0.1-μm metagenome contained higher numbers of non-identical sequence subtypes compared to the hypoxic water metagenome, with 19 and 6 hits to the amoA gene locus (encoding the active subunit of the ammonia monooxygenase enzyme) in the deep and hypoxic water metagenomes, respectively (Figure 4A).

Results from the alignment of 16S rRNA hits (dereplicated to <97% sequence identity) to the *N. maritimus* 16S rRNA gene were consistent with the amoA protein recruitment data (Figure 3B), indicating that close relatives of *N. maritimus* from both the deep ocean and hypoxic water habitats are capable of ammonia oxidation. Based on the maximum alignment depth, the number of 16S rRNA OTUs was estimated as 23 and 5 in the deep water and hypoxic water metagenomes, respectively (Figure 4B). When normalized by the total number of corresponding MGI 16S rRNA hits, the resultant OTU values indicated that the diversity of *N. maritimus*-like organisms was ~50% higher in the deep ocean compared to the hypoxic water habitat.

#### Bacteria

**Figure 5:**
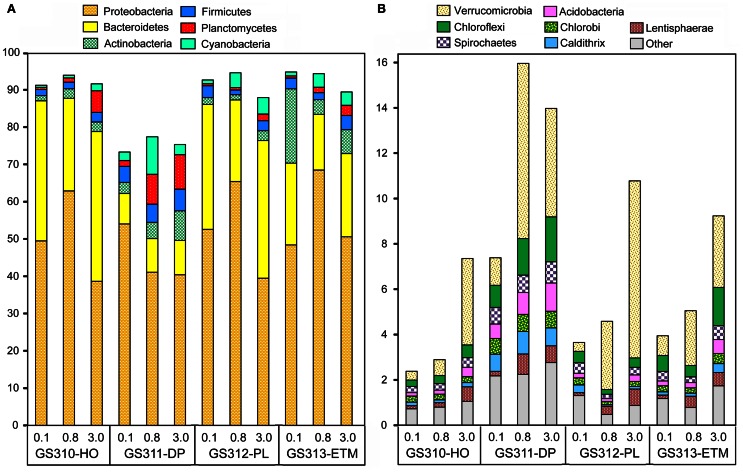
**Taxonomic composition of the domain Bacteria sequences in metagenomes.** The abundance of a phylum in a size fraction was calculated as the percentage of all corresponding hits (with ≥30% sequence identity over >70% of the alignment length) to the total annotated prokaryotic peptides. Sample names are encoded as described in the legend for Figure 3. **(A)** Most abundant phyla with values >5% of total post-QC reads in at least one metagenome; **(B)** Next-most abundant 10 phyla with values >0.1%.

Across all 12 metagenomes, the majority of classified peptides was always attributed to Bacteria. A similar analysis of taxonomic abundance was performed using SSU rRNA gene hits that were annotated separately from peptides (Figure 1B). Prokaryotic sequence representation was fairly similar with the two approaches (Figure 1B), particularly in the 0.1- and 0.8-μm size fractions, which contained >50% of prokaryotic sequences. Analysis of predicted bacterial peptides at ≥30% sequence identity revealed that the two most abundant phyla, Proteobacteria and Bacteroidetes, constituted from 50 to >85% of annotated prokaryotic peptides across all habitats (Figure 5A). Habitat-specific patterns were observed for some of the less abundant phyla, for example, Actinobacteria were enriched in the ETM 0.1-μm size fraction, whereas Planctomycetes were enriched in 3-μm fractions of low-oxygen habitats (Figure 5). Interestingly, the highest proportion of Cyanobacteria was observed in the deep water 0.8-μm size fraction; totaling 8% of all predicted prokaryotic peptides and annotated as Synechococcaceae at ≥60% sequence identity. The most plausible explanation for the presence of these photoautotrophs in the deep water is their origination in the euphotic zone and transport to the ocean floor as settling particles (Gooday et al., 1990).

**Figure 6:**
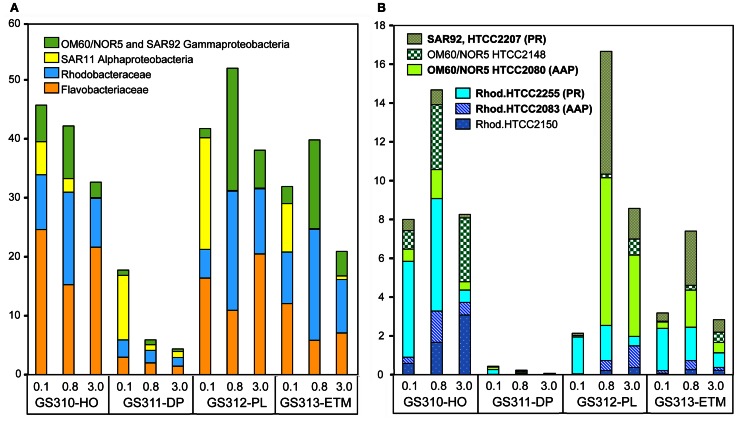
**(A)** Four most abundant (>10% in at least one metagenome) bacterial taxa detected in the metagenomes. Taxon abundance was calculated as percentage of hits with >60% identity (over >70% of the alignment length) to the total annotated prokaryotic peptides in the corresponding metagenome. **(B)** Abundance of Gammaproteobacteria from the marine group OM60/NOR5 and SAR92, and Alphaproteobacteria from the family Rhodobacteraceae (Rhodobacterales spp.) at sequence identities of >90% (over >70% of the alignment length). Genera shown in bold correspond to reference genomes containing genes associated with photoheterotrophy (PR, proteorhodopsin; AAP, aerobic anoxygenic photosynthesis). Sample names are encoded as described in the legend for Figure 3.

High-resolution taxonomic analysis for bacterial family (≥60% identity) and genus (≥90% identity) annotations elucidated the four most abundant taxonomic groups: (1) Flavobacteriaceae (Bacteroidetes/Flavobacteria), (2) marine roseobacters, including Rhodobacterales spp. of the Rhodobacteraceae family (Alphaproteobacteria), (3) SAR11 clade (Alphaproteobacteria), and (4) marine Gammaproteobacteria belonging to the OM60/NOR5 and SAR92 clades. Altogether, these groups constituted up to 50% of all annotated prokaryotic peptides (Figure 6A). In the CR coastal margin, they were most abundant in the three euphotic zone samples, ETM, plume, and hypoxic water, and underrepresented or absent in the deep water metagenomes, with the exception of the SAR11 clade in the 0.1-μm size fraction (Figure 6A).

**Figure 7:**
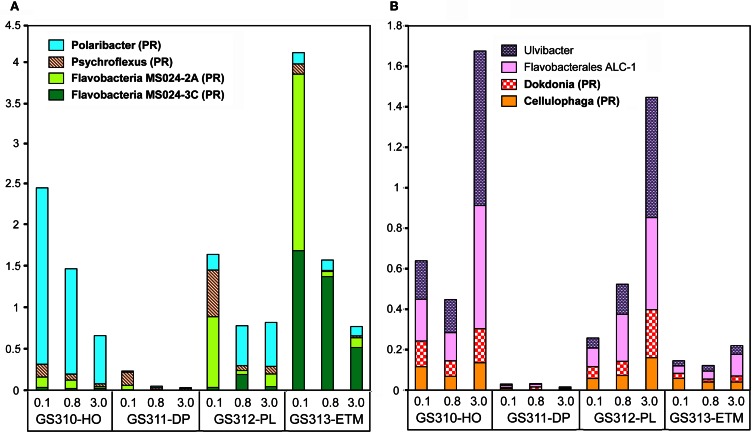
**Taxonomic composition at the genus/species level in the family Flavobacteriacea.** Taxon abundance was calculated as the percentage of all hits with >90% identity (over >70% of the alignment length) to the total annotated prokaryotic peptides in the corresponding metagenome. **(A)** Taxa particularly enriched in 0.1-μm size fractions. **(B)** Taxa particularly enriched in the 3-μm size fractions of algal bloom-associated metagenomes. Genera shown in bold correspond to reference genomes containing genes associated with photoheterotrophy (PR, proteorhodopsin; AAP, aerobic anoxygenic photosynthesis). Sample names are encoded as described in the legend for Figure 3.

Analysis of peptide annotations at ≥90% sequence identity revealed 16 well-characterized genera/species that were each represented with >1000 peptide hits: eight Flavobacteriaceae (Figure 7), four Rhodobacteraceae (the three most abundant taxa from this group are shown in Figure 6B), and four unclassified Gammaproteobacteria (three of which are shown in Figure 6B). In some cases these organisms appeared to be more marine (Polaribacter, and Psychroflexus) or estuarine (Gammaproteobacterium SAR92/HTCC2207, Flavobacteria spp. MS024-2A and MS024-3C) in their distribution (Figure 7). While most were enriched in the small size fractions of the euphotic zone samples, according to their cell sizes [0.1-μm for most Flavobacteriaceae (Figure 7A) and 0.8-μm for Rhodobacterales spp. (Figure 6B)], three Flavobacteriaceae, including the genera Ulvibacter, Dokdonia and Cellulophaga, and the unclassified Flavobacterales sp. ALC-1, were enriched in 3-μm particulate size fractions (Figure 7B).

#### Abundant photoheterotrophic bacteria in euphotic zone metagenomes

Twelve of the 16 most-abundant bacterial taxa, described above as enriched in the euphotic zone samples, corresponded to organisms with a photoheterotrophic lifestyle (shown in bold lettering in Figures 6B, 7). Ten of the reference genomes contained orthologs of proteorhodopsin (PR) (Stepanauskas and Sieracki, 2007; Newton et al., 2010), and two had genes for aerobic anoxygenic photosynthesis (AAP) (Ritchie and Johnson, 2012). AAP and PR-containing bacteria are most abundant in the coastal ocean (Yutin et al., 2007) in areas with high chlorophyll content, a possible consequence of their dependence on DOM leaking from dead phytoplankton cells (Ritchie and Johnson, 2012).

Protein recruitment plot analysis indicated that the euphotic zone metagenomes contained multiple high identity (≥90%) peptide hits to regions containing PR genes in Rhodobacterales sp.HTCC2255 and OM60/NOR5 Gammaproteobacterium sp. HTCC2080 genomes, and to marker genes for AAP, such as HTCC2083 pufM gene, and HTCC2080 bacteriochlorophyll a synthase (data not shown). Comparative analysis of functional gene categories indicated that rhodopsin gene hits were present almost exclusively in the three euphotic zone samples, and enriched particularly in 0.1- and 3-μm size fractions to ~1 hit per average bacterial genome equivalent. This pattern was different from that for RuBisCO, which was detected in all size fractions of all metagenomes, including those from the deep ocean, although it was generally most abundant in the 3-μm size fractions (data not shown).

#### SAR11 niche specialization

**Figure 8:**
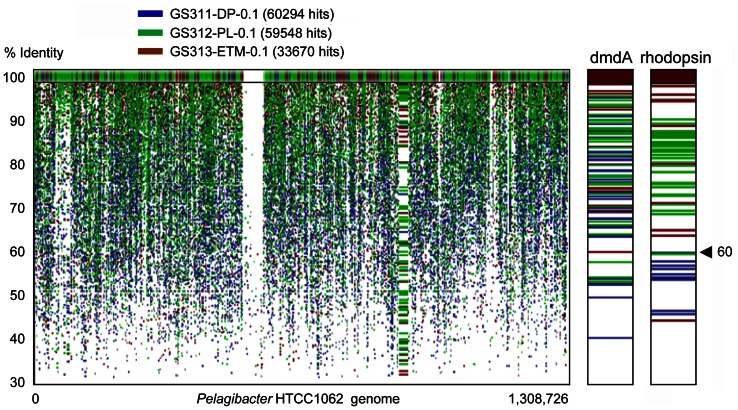
**Protein recruitment plot results showing metagenome peptide hits (with >30% identity over >70% of the alignment length) aligned against homologous loci of the *Pelagibacter ubique* HTCC1062 reference genome.** The X-axis shows the hit position along the *P. ubique* chromosome, and the Y-axis indicates the percent identity of a given sequence alignment for a metagenome hit and the reference locus, ranging from 30 to 100%. The upper panel displays the hits with 100% sequence identity. Metagenome hits from the plume, deep, and ETM 0.1-μm size metagenomes are shown in green, blue and brown, respectively. Panels to the right show magnified section of the recruitment plot with the *P. ubique* genes encoding SAR11_0246, dimethylsulfoniopropionate demethylase (dmdA, positions 248,657–249,766), and SAR11_0625, bacterial rhodopsin gene (positions 614,302–615,069).

The cosmopolitan SAR11 clade (Alphaproteobacteria) was abundant in all habitats. These organisms appeared to be almost exclusively free-living, corresponding to as much as 20% of all annotated peptides in the 0.1-μm size fractions, including the deep ocean bottom sample (Figure 6A). Analysis of 16S rRNA hits annotated as SAR11/Pelagibacter showed the presence of 5–7 OTUs in most habitats. Protein recruitment plots of the 0.1-μm metagenomes with the sequenced HTCC1062 reference genome (Figure 8) revealed uniform and uninterrupted tiling over almost the entire length (with the exception of rRNA operons intentionally excluded, i.e., the large gap in the middle of the plot). Closer analysis of specific genetic loci, i.e., the genome region containing the dimethylsulfoniopropionate demethylase gene [dmdA, SAR11_0246 (Varaljay et al., 2012)] showed multiple high identity hits across 0.1-μm size fractions of all metagenomes (Figure 8). In contrast, high identity (≥60 and ≥90%) hits to the Pelagibacter bacteriorhodopsin gene region [SAR11_0625 (Giovannoni et al., 2005a)] were found exclusively in the euphotic zone samples, and the total number of these hits was about half that for the dmdA gene region (Figure 8).

### Size fractionation assessment and metagenome comparison of particulate and free-living fractions

**Figure 9:**
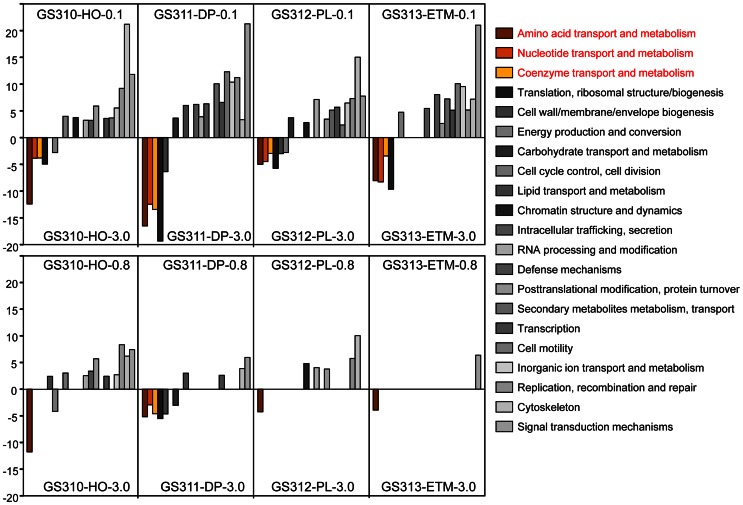
**Relative abundance of functional gene categories in the metagenomes expressed as D-rank values (bar graphs) calculated for 0.1-μm (upper panel) or 0.8-μm (lower panel) size fraction from each habitat compared to the corresponding 3-μm size fraction.** For each comparison, only significant D-ranks (>2.33, with *P*-values < 0.009) were used as measurements of overrepresentation (positive D-rank) or underrepresentation (negative D-rank). Functional gene categories related to transport of amino acids, nucleotides and coenzymes are colored brown and yellow.

We used the D-rank approach (Markowitz et al., 2008) to calculate normalized abundances and compare broad functional peptide categories based on COG annotations in each sample (Figure 9). Significant D-ranks for a COG category (>2.33, with *p* < 0.009) indicated over- (positive D-rank) or under- (negative D-rank) representation in the 0.1- or 0.8-μm metagenome compared to the corresponding 3-μm metagenome. By this measure, the 0.1-μm size fraction in all samples differed more from the corresponding 3-μm size fractions, while the 0.8-μm and 3-μm size fractions were relatively similar (Figure 9). Furthermore, three broad functional categories were consistently underrepresented in all 0.1-μm relative to 3-μm metagenomes, including amino acid, nucleotide and coenzyme transport. These functional categories are considered indicative of DOC utilization in coastal habitats (Poretsky et al., 2010; Rinta-Kanto et al., 2012).

**Figure 10:**
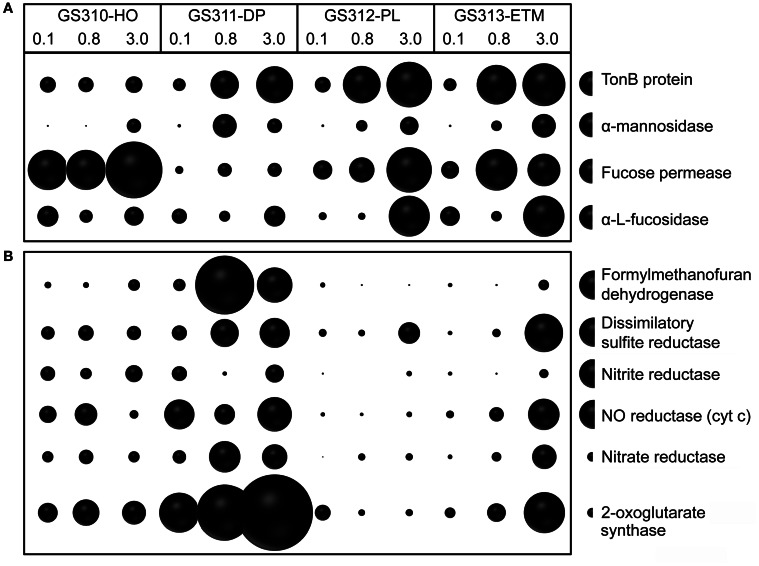
**(A)** Functional gene categories related to phytoplankton bloom utilization and dissolved organic matter assimilation, including TonB protein (COG0810), α-mannosidase (EC:3.2.1.24), fucose permease (COG0738), and α-L-fucosidase (EC:3.2.1.51). **(B)** Enrichment of genes involved in anaerobic pathways in ETM particles and the deep water metagenomes: formylmethanofuran dehydrogenase (COG1029, 1153, and 1229); dissimilatory sulfite reductase (desulfoviridin, COG2221 and 2920); nitrite reductase (nirS, nirK, EC:1.7.2.1); nitric oxide reductase (cyt c) (norB, norZ, EC:1.7.99.7); nitrate reductase (EC:1.7.99.4); and 2-oxoglutarate synthase (EC:1.2.7.3). Abundance for a functional gene category was calculated as the number of hits to a given category normalized by average bacterial genome equivalents in the corresponding metagenome. Abundance values are shown by bubble width for each size fraction in each sample. Half-bubbles to the right of each row show the scale and correspond to 1 gene per effective bacterial genome equivalent. Sample names are encoded as described in the legend for Figure 3.

In addition to broad COG categories, we analyzed specific functional gene groups that have been linked to successive decomposition of phytoplankton blooms in the North Sea (Teeling et al., 2012). These included TonB-dependent transporters and the carbohydrate-active enzymes α-mannosidase, α-L-fucosidase, and L-fucose permease, the latter three involved in assimilation of fucose, a major constituent of diatom exopolysaccharide (Teeling et al., 2012). In most cases, these categories were overrepresented in the 0.8- and 3-μm size fractions relative to the 0.1-μm size fractions (Figure 10A).

### Habitat differentiation of bacterial families

#### Clustering analysis of enrichment patterns

**Figure 11:**
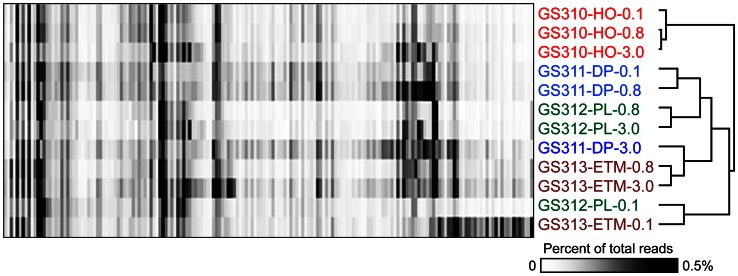
**2D Hierarchical clustering (complete linkage) of bacterial families (X-axis) in metagenomes (Y-axis).** In total, 117 bacterial families representing ≥0.1% of all annotated prokaryotic peptides (with ≥60% sequence identity over >70% of the alignment length) in at least one metagenome were selected for analysis. For each family, abundance was calculated as the percentage of all corresponding hits to total annotated prokaryotic peptides in the corresponding metagenome. Abundance values are shaded from white to black on a scale indicating percentages from low to high. Sample names are composed of the GS number, habitat (HO, hypoxic water; DP, deep ocean bottom; PL, plume; ETM, estuarine turbidity maxima), and size fraction: 0.1, 0.1–0.8 μm; 0.8, 0.8–3 μm; 3, 0.8–200 μm.

Despite being dominated by the four most abundant taxonomic groups described above, the domain Bacteria showed a diversity of less abundant taxa across the 12 metagenomes (Figure 5). This diversity was analyzed to characterize habitat-specific differences in the microbial communities. Abundance values for the bacterial families present in the samples (excluding the four most abundant taxa) were calculated using predicted peptide annotation data at ≥60% sequence identity. In total, 117 bacterial families were selected for analysis based on their occurrence at > 0.1% abundance (calculated as % of hits with ≥60% sequence identity for all annotated prokaryotic peptides) in at least one metagenome. Unsupervised 2D hierarchical clustering of these families within the metagenomes is shown in Figure 11. The two particulate size fractions from each metagenome, 3- and 0.8-μm, typically grouped together (except for the deep ocean sample). Interestingly, the free-living 0.1-μm fractions of the plume and ETM habitats (GS313-ETM-0.1 and GS312-PL-0.1) also grouped together (Figure 11), even though the salinity of these samples was quite different, as well as sample collection locations and times (~9 km and 10 days apart, Table 1). This may be due to the detection of common and temporally stable water column microbiota of the estuary-to-plume continuum in both samples.

Clustering of bacterial families based on similarity of their abundance patterns across size fractions and habitats (Figure 11) yielded four major clusters, altogether comprising 94% of the analyzed families (110 out of 117). The clusters included: (A) 12 families enriched in the smaller (0.1- and 0.8-μm) size fractions of the ETM and plume samples; (B) 27 families enriched in the ETM specifically, mainly occurring in 0.1-μm size fraction; and (C) and (D), 14 and 57 families, respectively, enriched in all deep ocean metagenomes, and in the ETM 3-μm size fraction. The C pattern corresponded to bacterial families that were more enriched in the ETM relative to the deep ocean, whereas the D pattern showed greater enrichment in the deep ocean relative to the ETM (and some corresponding enrichment in the hypoxic water 3-μm size fraction as well). Minor patterns were also observed, for example, specific enrichment of Ruthia-like bacteria from the SUP05 cluster (GSO–EOSA-1/ARCTIC96BD-19, Gammaproteobacteria) was observed in the hypoxic and deep ocean metagenomes (mainly in the 0.1- and 0.8-μm size fractions, data not shown). Association of these microorganisms with oxygen minimum zones has been described previously (Williams et al., 2012). We chose the three predominant patterns, B, C, and D (comprising 83% of all families analyzed) for comparisons of genus/species (≥90% sequence identity) distributions across size fractions and habitats.

#### Riverborne bacterial enrichment pattern

**Figure 12:**
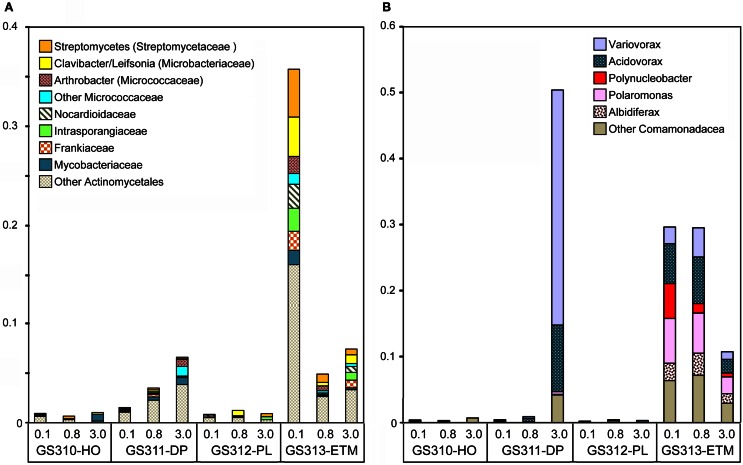
**Bacterial taxa enriched mainly in the ETM metagenomes. (A)** Genera and families from the order Actinomycetales (Actinobacteria). **(B)** Genera from families Burkholderiaceae and Comamonadaceae (Betaproteobacteria). Taxon abundance was calculated as the percentage of all corresponding hits with >90% identity (over >70% of the alignment length) to total annotated prokaryotic peptides. Sample names are encoded as described in the legend for Figure 3.

Twenty-seven bacterial families were highly enriched in the ETM 0.1- and 0.8-μm size fractions. Of these, the most abundant genera with ≥90% sequence identity (Figure 12A) corresponded to the order Actinomycetales (Actinobacteria). These were common soil taxa, found in the rhizosphere (where they participate in the organic matter decomposition), or in terrestrial plant biomass (as pathogens), but not in marine habitats (such as the marine Actinobacteria Salinispora). Three other genera were from Burkholderiaceae and Comamonadaceae (Betaproteobacteria, Figure 12B): Polynucleobacter, believed to be an exclusively freshwater organism, and Polaromonas and Albidiferax, that have mostly been recovered from glacial sediments, ice and snowmelt (Darcy et al., 2011). The ETM enrichment pattern of these genera (Figure 12) suggested that they were likely free-living (0.1 and 0.8-μm fractions), allochtonous, and advected into the CR estuary with the freshwater influx.

Interestingly, two other Betaproteobacteria, Variovorax and Acidovorax, in addition to being enriched in the 0.1- and 0.8-μm ETM metagenomes (Figure 12B) were also abundant in the 3-μm size fraction of the deep ocean habitat (Figure 12B). Variovorax species appear to be ecologically diverse, having been described as ubiquitous, aerobic soil bacteria involved in the rhizosphere sulfur cycle through utilization of sulfonates (Dul'tseva et al., 2012), identified in deep ocean sediments (1340 m) of the South China Sea, and within the human oral cavity [as reviewed in Wang and Gu (2006)].

#### Co-enrichment of taxa in deep water and ETM particles provides evidence for anoxic microzones

**Figure 13:**
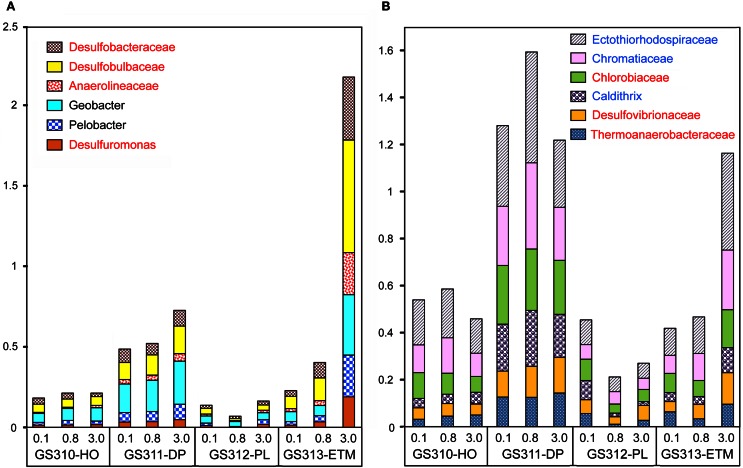
**Enrichment of anaerobic taxa in the deep ocean and in ETM 3-μm metagenomes.** The abundance of a family/genus in a size fraction was calculated as the percentage of all corresponding hits with >60% identity (over >70% of the alignment length) to total annotated prokaryotic peptides. **(A)** Genera and families more abundant in the ETM metagenome than in the deep water samples. **(B)** Genera and families that are enriched to a similar degree in the deep ocean and ETM metagenomes. Taxa shown in red lettering are characterized as strict anaerobes. Taxa shown in blue lettering are characterized as denitrifying bacteria. Sample names are encoded as described in the legend for Figure 3.

Seventy-one of 117 analyzed bacterial families (at ≥60% sequence identity) were specifically enriched in the ETM 3-μm particulate fraction, being 2 to 10X more abundant (*p* < 0.001) in comparison with the 0.1- and 0.8-μm metagenomes from this habitat (Figure 13). These families were also enriched in the three metagenomes from the deep ocean sample (Figure 13). Four of the families identified were the sulfate reducing bacteria Desulfobacteraceae, Desulfobulbaceae, Desulforomonas, and Desulfovibrionaceae (Figure 13). Sulfate reducers utilize H2 and acetate produced during the final steps of complex organic matter remineralization, and because of this can have a direct impact on microbially-driven methanogenesis, through competition for these substrates (Reeburgh, 2007; Conrad, 2009). Previous analysis of 16S rRNA described the presence of archaeal methanogens in the CR estuary (Crump and Baross, 2000a), and we also found sequences related to methanogenic archaea (i.e., families Methanosarcinaceae, Methanomicrobiaceae, and Methanobacteriaceae) enriched in the ETM 3-μm size fraction, albeit to a much lesser extent in comparison with their enrichment in the deep water and in the plume (Figure 3A).

For at least 10 identified families, members are considered to be either strict anaerobes, e.g., Anaerolineaceae and Chlorobiaceae, or facultative anaerobes, such as two Mn/Fe-reducing genera commonly found in anoxic and brackish sediments, Pelobacter and Geobacter (Figure 13). Previously, many of these taxa were described in the CR estuary, including active Mn/Fe reducing bacteria associated with ETM (Bräuer et al., 2011), and anaerobic, photoautotrophic green sulfur bacteria, Chlorobi (Crump et al., 1999). We analyzed the abundance of several genes consistent with an anaerobic lifestyle: (1) 2-oxoglutarate synthase (EC:1.2.7.3), the key enzyme of the reverse TCA cycle; (2) formylmethanofuran dehydrogenase (COG1029, 1153, and 1229) specific for methanogenesis; and (3) dissimilatory sulfite reductase (desulfoviridin, COG2221 and 2920) involved in anaerobic sulfate reduction. Relative abundance of all these genes was increased in all deep water, and in the ETM 3-μm metagenomes (Figure 10B).

Furthermore, functional gene categories involved in dissimilatory nitrate reduction were enriched in the deep water and in the ETM 3-μm size metagenomes (Figure 10B). In support of this observation, three families enriched in metagenomes from ETM particulate fractions and the deep ocean (Figure 13B, blue lettering) contain members capable of dissimilatory nitrate reduction, which also occurs in anoxic and low-oxygen environments. Our previous study using oligonucleotide DNA microarrays showed that the average expression levels of genes involved in denitrification and dissimilatory nitrate reduction to ammonia pathways were 1.5 to 2X higher in samples from the estuary in comparison to samples from freshwater and the coastal ocean. Taken together, the data indicate that particle-attached microbiota may perform dissimilatory nitrate reduction in the CR system, similar to reports for the particle-attached microbiota in the River Rhone plume and coastal waters of the northwestern Mediterranean Sea (Omnes et al., 1996; Michotey and Bonin, 1997).

## Discussion

This first examination of microbial metagenomes from four diverse habitats within the CR coastal margin provides metagenome-scale evidence for diverse communities of particle-attached and free-living microbial populations in the water column. Our data suggest that the 0.8- and 3-μm size fractions of each sample were enriched for particle-attached microorganisms and large eukaryotic plankton, while the 0.1-μm size fractions were mostly representative of free-living bacteria and archaea. Compared to the corresponding 0.1-μm size fraction, relatively higher similarity was observed between the 0.8- and 3-μm size fractions of each sample with respect to GC content, effective genome size (Table 1), general taxonomic composition (Figure 1A), and functional gene categories (Figure 10). This result was particularly notable in the ETM and plume, presumably due to higher amounts of particulate matter in these samples.

Eukaryotic plankton composition varied greatly across habitats. In all three euphotic zone samples marine diatoms dominated phytoplankton taxa, with Cryptophyta observed in the ETM sample (Figure 2). The cryptophyte algae are ingested by dinoflaggelates and the ciliate *M. rubrum*, the latter retaining the chloroplasts and nuclei in order to maintain an autotrophic lifestyle (Herfort et al., 2011a). A large bloom of *M. rubrum* was observed in the estuary around the same time and within a few km of the ETM sampling location (Herfort et al., 2011a). Thus, the Cryptophyta enrichment may correspond to both free-living algae and organelles contained within *M. rubrum* cells. Unfortunately, the lack of *M. rubrum* genome sequence information precluded further analysis. Our results suggest that variations in the particle-attached microbial assemblages may be determined by habitat-specific sources of organic matter originating from dead or senescent eukaryotic cells, the composition of which impacts the local detrital food web and subsequent heterotroph growth and activity (Ploug et al., 1997, 2008).

The free-living microbiota in the deep water and hypoxic habitats contained an abundance of marine ammonia-oxidizing Thaumarchaeota of the MGI group (Figure 3). Our data were consistent with other studies demonstrating that, in the eastern Pacific Ocean, MGI archaeal abundance was: (1) highest below the euphotic zone, (2) reduced to near absence in the surface layers of the water column, particularly in summer, and (3) inversely correlated with chl a concentrations (Mincer et al., 2007; Church et al., 2010; Stewart et al., 2012).

The apparent abundance of methanogens observed in the plume sample was surprising (Figure 3A). This result may be explained by previous reports (Marty, 1993; van der Maarel et al., 1999) suggesting that the presence of methanogens in oxygenated surface waters is due to their expulsion from the digestive tracts of zooplankton in fecal pellets, implying that subsequent pellet re-suspension may have led to their enrichment in the smaller size fraction of the CR plume.

Bacteria dominated the microbial communities of all analyzed habitats (Figure 1). The most abundant taxonomic groups included the family Flavobacteriaceae, marine roseobacters of the Rhodobacteraceae family, SAR11 clade Alphaproteobacteria, and marine Gammaproteobacteria of the OM60/NOR5 and SAR92 clades (Figure 6). Previously identified as major taxa comprising marine heterotrophic bacterioplankton, these organisms are frequently found associated with organic particles, but also appear to represent a significant portion of the free-living microbial assemblage in nutrient-rich microenvironments (Bauer et al., 2006; Woebken et al., 2007; Gonzalez et al., 2008). The analyzed metagenomes contained close relatives of these well-characterized taxa with thousands of predicted peptide hits at >90% identity. Twelve of the 16 most-abundant bacterial taxa corresponded to organisms with a photoheterotrophic lifestyle, using both organic substrates and light energy for growth and survival. They were highly enriched in the euphotic zone metagenomes, including the estuary, plume and hypoxic coastal ocean, with up to 20% of the total annotated prokaryotic peptides corresponding to the described genera, and containing multiple high-identity (≥90%) hits to marker genes (PR, bacteriochlorophyll a synthase). Our data are consistent with previous observations that photoheterotrophs are the most abundant bacteria in the coastal euphotic ocean (Yutin et al., 2007; Ritchie and Johnson, 2012).

Interestingly, some studies have reported an association of AAP bacteria with particles (Waidner and Kirchman, 2007; Lami et al., 2009; Cottrell et al., 2010). This relationship appears to vary with environmental conditions in different habitats. Our data indicate that in the CRCM, AAP bacteria were generally more enriched in the particulate (0.8–3.0 and 3–200 μm) compared to free-living (0.1–0.8 200 μm) size fractions, while PR-containing bacteria were relatively more abundant in the two smaller size fractions (Figures 6B, 7A). This association may be particularly important in the CR estuary, where the hydrodynamics of the system extends the residence time of particle-attached bacteria dramatically, compared to free-living microorganisms which are washed out much more quickly (Crump et al., 1999; Crump and Baross, 2000b).

In addition, the bacteriorhodopsin gene region [SAR11_0625 (Giovannoni et al., 2005a)] from abundant Pelagibacter was found exclusively in euphotic zone samples, suggesting niche specialization of SAR11 clade members. Previous results indicate that genetic diversity of the SAR11 clade correlates to niche specialization (Field et al., 1997). For example, coastal isolates have glycolysis-related genes for glucose utilization, while open-ocean isolates do not (Schwalbach et al., 2009). Since the rhodopsin gene is unlikely to be functional in the deep ocean, it may be lost in SAR11 inhabitants due to a genome “streamlining” process (Giovannoni et al., 2005b).

Although microbial taxa were enriched in the size fractions corresponding to their cell sizes, both predicted eukaryotic peptides (Figure 1A) and chloroplast SSU rRNA (15–43% of total reads) were observed in all size fractions (with the exception of GS311-DP-0.1, Figure 1B). The smallest free-living marine eukaryote, the green algae Ostreococcus (Prasinophytes, Chlorophyta), is 0.95 μm in size (Courties et al., 1994), and living cells would not be expected to pass through the 0.8-μm filter. Our finding of Chlorophyta DNA in the 0.1-μm fractions is therefore most likely explained by leakage from dead and decaying cells and subsequent persistence in the environment. Similar results were obtained previously in a study comparing estuarine samples collected with or without pre-filtering through a 1-μm screen: both sample types showed remarkable similarity, and contained rRNA genes from chloroplasts of eukaryotic phytoplankton (Crump et al., 2004). In addition to the apparent leakage of DNA-containing decaying biomass from larger to smaller size fractions, we also observed the particle aggregation and flocculation resulting in sequences from cells small enough to pass through the 0.8- and 3-μm filters appearing in the larger size fractions. Regardless of some apparent cross-contamination among fractions, results from using the D-rank approach (Markowitz et al., 2008) to compare metabolic properties across fractions were consistent with particle “hotspots” in the larger fractions for POC degradation, subsequent DOC utilization, and active uptake through the TonB-dependent transport system.

Particulate fractions of the plume and hypoxic water samples were enriched with sequences from Flavobacteracea spp previously found in association with phytoplankton. For example, the genus Ulvibacter was initially isolated from a member of the green marine algae (Nedashkovskaya et al., 2004), and its abundance increased dramatically in the presence of coastal diatom blooms in the North Sea (Teeling et al., 2012). Both hypoxic water and plume 3-μm metagenomes with abundant Ulvibacter sequences also had significant numbers of hits to diverse algae (Figure 2). Flavobacteria are known to be particularly abundant during periods of high primary production (Williams et al., 2012), and many of them are associated with phytoplankton particles and degrade polysaccharides (Kirchman, 2002; Gonzalez et al., 2008; Teeling et al., 2012). It therefore seems possible that the other three organisms with enrichment patterns similar to Ulvibacter may also be associated with diatom particulate material during blooms. Another possibility is the involvement of some of these Flavobacteria in the degradation of macroscopic algae, such as the Phaeophyta kelps (brown algae), which are members of the order Laminariales. Extensive beds of large subtidal kelps are typical of the temperate Pacific Northwest coastline, they generate large quantities of water-borne particles and dissolved organic compounds that support distinctive communities of prokaryotes (Schapira et al., 2012). Genomes of many Flavobacteria, including the Flavobacterales sp. ALC-1, contain gene clusters that are involved in degradation of alginate, the main component of the kelp cell wall (Thomas et al., 2012).

Differences between the free-living and particle-attached microbiota were especially prominent in the CR estuary. None of the bacterial genera enriched in the ETM 0.1-μm size fraction were prevalent in the corresponding 3-μm particulate fraction (Figure 12). Many of the free-living bacteria corresponded to freshwater and soil taxa, and were apparently riverborne and advected into the estuary. Consistent with the previous findings (Crump et al., 1999, 2004; Crump and Baross, 2000b), the particle-attached microbial community of the estuary was phylogenetically distinct from the free-living ETM assemblage, instead sharing similarities with the deep ocean bottom community (Figure 13). The ETM particles were enriched for anaerobic and microaerophylic bacterial populations, and the corresponding 3-μm ETM metagenome contained hits to key enzymes involved in anaerobic metabolisms (Figure 10B).

The majority of bacterial taxa enriched in ETM particles and the deep water habitat were not abundant in the plume 3-μm size fraction, or in the hypoxic water fractions (with oxygen concentrations comparable to those of the deep ocean) (Figure 13). Our data therefore showed no evidence for direct transport of particulate matter from the estuary to the deep ocean through the CR plume and euphotic coastal ocean. Furthermore, the eukaryotic sequence data suggested that particulate compositions varied significantly among habitats, reflecting different particle sources (Bauer et al., 2006). The deep water particulate matter contained an abundance of marine animal sequences (Figures 1B, 2), whereas the euphotic zone samples contained mostly algal DNA (Figure 2). In the ETM, an additional source of fresh autochthonous particulate organic matter was the *M. rubrum* bloom (Herfort et al., 2011b), and possibly, organic matter-rich sediments from the CR lateral bays (Sherwood et al., 1990). Still, a common source for “seeding” of the various habitats with anaerobic taxa could not be ruled out, as the predicted peptide annotations used here could not unequivocally establish whether the same subtypes populated different habitats, or if they were related, but distinct members of the same family/genus. In any case, the degree of enrichment observed for these microorganisms in the ETM and deep water (up to 4% of all annotated prokaryotic peptides, Figure 13) suggests cellular activity and proliferation in both habitats. In support of this, previous observations of an Mn anomaly associated with ETM in the CR estuary suggested biological activity of anaerobes in the form of organic carbon oxidation by microbes that use Mn oxides as electron acceptors, presumably in the suboxic interiors of ETM particles, and was reported to account for over 16% of riverine Mn (Klinkhammer and McManus, 2001).

Anoxic microzone formation in ETM particles may be explained by activities of aerobic heterotrophic microorganisms responsible for degradation of particulate matter and subsequent release of DOC (Baross et al., 1994). Particle-attached microbes have enhanced rates of cellular activity compared to unattached bacteria (Crump et al., 1998), and their high respiration rates in nutrient-rich aggregates can lead to steep oxygen gradients forming inside particles (Woebken et al., 2007; Ploug et al., 2008). This process may select for facultative anaerobes in the otherwise oxygen-replete estuary. In the tidally-influenced estuary, association of bacteria with ETM particles may additionally help to mitigate the effects of changing salinity. Particle-trapping by ETM and microniche development may therefore be instrumental in maintaining viability of these organisms in the oxygenated water column at variable salinities. Understanding whether these organisms and their associated activities impact organic matter degradation in the estuarine water column is a topic of further research.

### Conflict of interest statement

The authors declare that the research was conducted in the absence of any commercial or financial relationships that could be construed as a potential conflict of interest.
